# Evaluation of genetic variability in the collared peccary *Pecari tajacu* and the white-lipped peccary *Tayassu pecari* by microsatellite markers

**DOI:** 10.1590/S1415-47572010005000002

**Published:** 2010-03-01

**Authors:** Roxane Wirschum Silva, Thales R. O. de Freitas, Ives José Sbalqueiro

**Affiliations:** 1 Departamento de Genética, Universidade Federal do Paraná, Curitiba, PRBrazil; 2 Departamento de Genética, Universidade Federal do Rio Grande do Sul, Porto Alegre, RSBrazil

**Keywords:** microsatellite markers, *Tayassu pecari*, *Pecari tajacu*, genetic variability, animals of captivity

## Abstract

In this study, the microsatellite technique was used to evaluate the genetic variability in populations of collared and white-lipped peccaries kept in captivity. Six primers developed for domestic pigs were used and amplified in both species. They revealed the presence of five polymorphic loci and one monomorphic locus. The polymorphic loci included 4 of the 16 alleles in collared peccaries, and 3 of the 10 alleles in the white-lipped peccaries. Polymorphic information content (PIC) in both species and all the loci was highly informative. The probability of paternity exclusion (PEC), if one of the parents is known, was almost as high in white-lipped peccaries (95.53%) as in the collared (99,48%). The *Fst* values for collared (0.042) and white-lipped (0.1387) peccaries showed that both populations are not structured. The *Fis* values for all loci, except ACTG2 in white-lipped peccaries (-0.0275) and in both species (0.1985 to 0.9284 in collared peccaries and 0.3621 to 0.4754 in the white-lipped), revealed a high level of homozygosis, probably caused by inbreeding. Data on heterologous amplification and genetic variability in collared and white-lipped peccaries are presented for the first time.

The family Tayassuidae belongs to the order Artiodactyla, suborder Suiformes, superfamily Suoidea, and contains three genera and three species (Groves and Grubb, 1993): the collared peccary *Pecari tajacu* (Linnaeus, 1758), the white-lipped peccary *Tayassu pecari* (Link, 1795) and the chacoan peccary *Catagonus wagneri* (Rusconi, 1930). *Pecari tajacu* has a geographical distribution from Arizona to Argentina, where it inhabits rain and xeric forests, as well as the desert, in groups of 5 to 50 animals. In contrast, *Tayassu pecari* occurs from Mexico to northern Argentina, occupying primary forest in groups of up to 400 individuals (Beck, 2006). Cytogenetic studies show that *T. pecari* has 26 chromosomes, the two largest chromosome pairs being submetacentric, besides nine pairs of bi-armed chromosomes, the smallest autosomal pair being acrocentric, whereas the sexual pair is formed by a medium-sized acrocentric X chromosome and a small acrocentric Y. The *Pecari tajacu* karyotype has 2n = 30, with eight autosomal pairs of bi-armed chromosomes and six autosomal acrocentric pairs, the X sex chromosome being medium submetacentric and the Y small acrocentric (Bosma *et al.*, 2004; Adega *et al.*, 2006). Although *P. tajacu* exhibits a stable karyotype, chromosomal gaps and breaks have been previously and very frequently observed in the autosomal chromosomes in the very same collared peccary population sampled for the present report (Lima *et al.*, 2004). According to these authors, a vermifuge based on ivermectin was the most likely cause of these chromosomal alterations.

In Brazil, both species are used as a source of leather and meat, their leather being used in fine goods, such as gloves and purses, thereby making their conservation of extreme importance (Deutsch and Puglia, 1990). Thus, there is the dire need for management and captive-breeding programs, as these wild animals require special care. Under appropriate conditions in captivity, their maintenance is a viable economic activity. Studies with molecular markers and microsatellites in the Tayassuidae are, as yet, few, due to the need for developing species-specific primers. Furthermore, as this kind of population is artificial and inbreeding on farms is high, the analysis of population genetic variability becomes imperative. Microsatellites are the best molecular markers for evaluating this variability, affording outstanding results. Nevertheless, this problem has already been partly solved through heterologous amplification of microsatellite loci of the domestic pig *Sus scrofa domestica*, for instance, in both *P. tajacu* (Lowden *et al.*, 2002; Gongora *et al.*, 2002) and *T. pecari* (Gonela, 2003). The two main goals of the present study were to evaluate genetic variability in both species, a completely unknown factor in captive populations on farms in the state of Paraná, by using six heterologous molecular microsatellite markers specifically developed for the domestic pig (*Sus scrofa domestica*).

Blood samples were collected from 45 captive collared peccaries on the Fazenda da Praia, the municipality of Tibagi (7 males and 5 females), the Parque Municipal das Araucárias, the municipality of Guarapuava (3 males and 2 females), and the Fazenda Experimental Gralha Azul, the municipality of Fazenda Rio Grande (14 males and 18 females). Blood was also collected from 25 captive white-lipped peccaries from the Parque Municipal das Araucárias (11 males and three females) and the Fazenda Experimental Gralha Azul (five males and six females) (Table S1). The three municipalities are located in the state of Paraná, Brazil.

DNA was extracted by the salting-out technique (Medrano *et al.*, 1990). The six loci used were SW1407, SW1408, SW857, SW2411, ACTG2, and SW444. The samples were first quantified in a spectrophotometer, so as to measure optical densities in ng/mL, and then diluted in sterile Milli-Q water to obtain samples of DNA of an approximate concentration of 20 ng/mL. The amplification conditions for each locus were the same for both species. During the amplification process itself, a unit of *Sus scrofa domestica* was used as positive control for the reaction. A 10% native (undenatured) polyacrylamide gel was used on 23 cm x 25 cm plates for electrophoresis of the amplified products. After electrophoresis, the gel was stained by a modified Tegelstrom (1992) method. The size of the alleles was determined by comparison with a 25 bp molecular weight ladder (Invitrogen).

Genetic variability was estimated by means of descriptive statistics with the GDA (Genetic Data Analysis) program (Lewis and Zaykin, 2001), thereby determining the number of polymorphic loci and alleles per locus, as well as observed (*Ho*) and expected (*He*) heterozygosity. Fisher's test was used to test shunting lines of the Hardy-Weinberg equilibrium in each of the two species of peccary, by the shuffle method (20,000 shuffles) using the GDA program (Lewis and Zaykin, 2001). *Fst* was estimated (Weir and Cockerham, 1984) was done by Analysis of Molecular Variance - (AMOVA; Excoffier *et al.*, 1992) - with the ARLEQUIN 3.0 program (Excoffier *et al.*, 2005). The *Fis* parameter (Wright, 1951) was obtained in accordance with the GDA program (Lewis and Zaykin, 2001), which was also used to calculate both the genetic distance between populations (Nei, 1972) and allele frequencies. A dendrogram was constructed and cophenetic correlation values calculated by means of distance values obtained with the aid of the NTSYSpc 2.1 (Rohlf, 2000) program and through the UPGMA method (Sneath and Sokal, 1973). Polymorphic information content (PIC) and probabilities of paternity exclusion were calculated using the CERVUS 2.0 (Marshall *et al.*, 1998) program.

Through the analysis of six markers, the presence of one monomorphic (SW1407) and five polymorphic loci in both species of peccary was revealed. The monomorphic locus showed fragments of 112 bp in *P. tajacu* and 114 bp in *T. pecari*. In *P. tajacu*, 51 alleles ranging from 103 bp (SW444) to 207 bp (SW2411) were found in the polymorphic loci (Table 1a), whereas in *T. pecari*, 29 different alleles ranging from 98 bp (SW444) to 228 bp (SW2411) were so found (Table 1b). Genotypic frequencies found in both *P. tajacu* and *T. pecari* can be seen in Figure 1.

The values of observed (*H*_*o*_) and expected (*H*_*e*_) heterozygosity for all loci in *P. tajacu* and *T. pecari* are shown in Table 1 (1a and 1b, respectively). In *P. tajacu*, observed heterozygosity values were significantly less than expected in all loci. Higher than expected heterozygosity was only encountered in locus ACTG2 in *T. pecari*. Hardy-Weinberg equilibrium was attained for loci after correction by the Bonferroni test.

The values obtained for polymorphic information content (PIC) were high in all the five loci in both species of peccary. The estimated values of the probabilities of exclusion of paternity (PE) in each locus and species, when neither of the parents is known (PE 1) or when only one is (PE 2), can be observed in Table 1. The most informative loci were ACTG2 in collared peccaries and SW2411 in the white-lipped peccaries. The probabilities of agreed exclusion (0.965 and 0.995 in collared, and 0.826 and 0.955 in white-lipped peccary) obtained through the five systems in the set were high in both *P. tajacu* (PEC 1 and PEC 2) and *T. pecari* (PEC 2).

The values of *Fst* for each locus and the totals, on considering the three population samples from collared peccaries and the two from white-lipped as one single population, can be observed in Table 1. The results of *Fst* (0.042 for collared and 0.1387 for white-lipped peccaries) indicate that there is no structuring, or, in other words, that genetic variability found within and not between the population samples indicates the existence of mutual gene flow. The *Fis* parameter values in *P. tajacu*, ranging from 0.199 to 0.814 in a total 0.543 (Table 1a), and in *T. pecari*, ranging from 0.362 to 0.475 in a total of 0.351 (Table 1b), are extremely high, except for the negative value at locus ACTG2 in the white-lipped peccary. These high values indicate an excess of homozygotes in the samples.

The *Fst* parameter was established by comparing the two species of peccaries, where the value of 0.2086 indicates structuring. As reported by Nei (1972), the genetic distances show a sufficiently evident separation between the two species, with the upper branch corresponding to the populations of collared peccaries, and the lower to those of the white-lipped peccaries (Figure 2).

The amplification of microsatellite regions by using primers from a certain species for similar species, is extremely laborious, as, in addition to a divergence in time, one may be dealing with different loci located on different chromosomes. However, there has been certain success with heterologous amplification involving microsatellites, such as studies undertaken by Gemmell *et al.* (1997) with pinnipeds, Slate *et al.* (1998) with deer and sheep, Lowden *et al.* (2002) with wild boars, Gongora *et al.* (2002) with peccaries, Gonela (2003) with swine and peccaries, and Lau *et al.* (2004) with monkeys. In the present study, the six primers originally developed for the domestic pig were satisfactorily amplified for *P. tajacu* and *T. pecari*, thus confirming the findings of Gongora *et al.* (2002) and Lowden *et al.* (2002) for locus SW857 in *P. tajacu* and of Gonela (2003) for loci ACTG2 and SW444 in *T. pecari*. In addition, the results presented here for loci SW1407, SW1408, and SW2411 in collared and white-lipped peccaries, besides SW857 in the white-lipped alone, and ACTG2 and SW444 in collared peccary, are reported for the first time.

With regard to the number of obtained alleles, when compared to the number of alleles described for the domestic pig, in *T. pecari* a reduction in the number of alleles was observed in all the loci (four in SW1408, one in SW1407, five in SW857, ten in SW2411, seven in ACTG2, and three in SW444). In *P. tajacu* this occurred only in loci SW1407, SW2411, and SW444 (one, four, and seven, respectively), whereas in the others there was an increase (11 in SW1408, 13 in SW857, and 16 in ACTG2). Gonela (2003), on working with heterologous primers, found that reduction from the original species can vary widely, and offered two explanations for this. The first is related to behavior, where individuals of one flock do not cross with those of another, thereby promoting endogamy and the founder effect. In the second, the reduction could be related to the presence of null alleles, thus posing which poses a problem when primers from one species are used in another. If the null alleles occur in homozygosis, this will be considered as an imperfection in the amplification reaction, whereas if they do occur in heterozygosis, the heterozygote will be considered as a homozygote.

In collared peccaries and considering each locus separately, the number of homozygous individuals increased, whereas observed heterozygosis was lower than expected, thus disclosing low genetic variability. In white-lipped peccaries, the situation proved to be similar, with the exception of locus ACTG2, where the value was higher than expected, an indication of excessive heterozygosity. In both the collared and white-lipped peccaries, the values found for polymorphic information content (PIC) were highly informative (Botstein *et al.*, 1980) for all the loci. In collared peccaries, exclusion probability (PEC) was high when neither of the parents is known (96.48%), likewise when only one is (99.48%), both results being expedient. In white-lipped peccaries, these probabilities were, respectively, 82.57% (low) and 95.53% (high), *i.e.*, none of them reached a desirable value. Therefore, in collared peccaries the set of five polymorphic loci revealed high potential for use in paternity or maternity tests. However, in white-lipped peccaries, one of the factors that possibly influenced the obtained probabilities was the number of observed alleles, as in the majority of loci this was lower than that observed in collared peccaries. Therefore, in this case we suggest the use of novel markers and an increase in sample size.

This is the first estimate regarding a paternity test carried out with peccaries. Such tests are important, in that studies are increasing in economically important herds, this including equines and bovines.

In both collared and white-lipped peccaries, a lack of structure was observed (*Fst* 0.042 and 0.1387, respectively), thereby indicating little differentiation between populations, mutual genetic flow, and that the existent genetic variability is within and not between populations. These values were expected, since the captive populations were artificial and recently formed, therefore with insufficient time for self-structuring. Nevertheless, when comparing the two species in relation to the five polymorphic loci, a high degree of structuring was found (*Fst* equal to 0.2086), thus indicating an intermediate division, which was also expected due to existing reproductive isolation.

The *Fis* index was high for collared peccaries in almost all loci (0.1985 to 0.9284), as for the white-lipped (0.3621 to 0.4754), thereby indicating excess populational homozygosity, mainly brought about by inbreeding. Several factors may be acting, either separately or together. Thus, the fact that these are captive populations, in relatively low effective numbers and recently formed (perhaps no longer than ten years previously), and constantly receiving new elements of uncertain origin, could, in a certain way, contribute to inbreeding. Moreover, these two species are listed as threatened in the state of Paraná (Mikich and Bérnils, 2004), mainly due to habitat deforestation. It is probable that the populations of these species of peccaries, subjected to such a strong selective pressure, have undergone a reduction in effective sizes, consequently favoring inbreeding or genetic drift. In addition to inbreeding, other sources may also be involved or equally responsible for the excess homozygosity observed in both species, such as errors in genotyping individuals, the presence of null alleles and the Wahlund effect. In the Wahlund effect, the genetic effect of isolation depends on the size of the isolated populations. Thus, if a population were divided into isolated parts, the result of isolation would be similar to that of consanguineous mating, that is, the frequency of homozygotes in the population would increase (Beiguelman, 1994).

**Figure 1:**
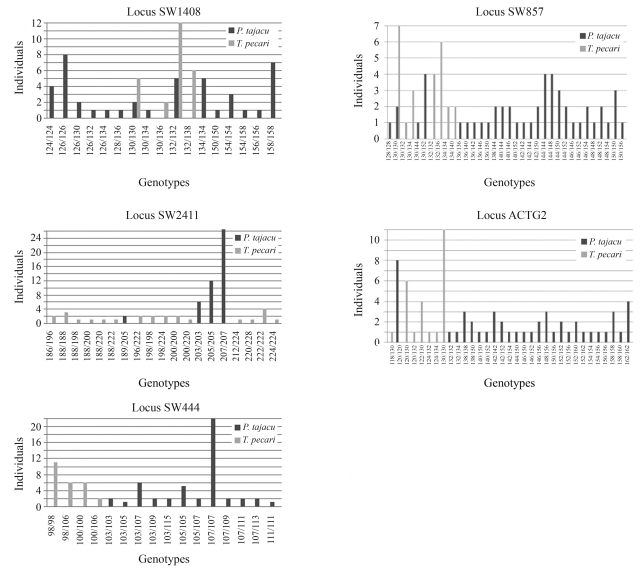
Graphs showing the number of individuals and genotypes found in the five loci analyzed.

**Figure 2:**
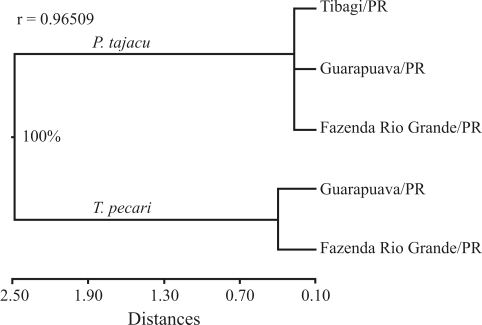
Representative dendrogram of the genetic distances between collared and white-lipped peccaries. The numbers of knots are the bootstrap values with 10,000 replications.

## Supplementary Material

The following online material is available for this article:

Table S1 Localities and identification of voucher specimens.

This material is available as part of the online article from http://www.scielo.br/gmb

## Figures and Tables

**Table 1:** Indices of genetic variability found in *Pecari tajacu* and *Tayassu pecari*.

Locus	N	NA	SIZE (bp)	Ho	He	P	PIC	PE1	PE2	Fis	Fst
(a) *Pecari tajacu*											
SW1408	43	11	124-158	0.163	0.866	n.s.	0.840	0.551	0.713	0.814	-0.003
SW857	49	13	128-156	0.714	0.889	n.s.	0.870	0.615	0.763	0.199	0.031
SW2411	48	4	189-207	0.042	0.576	n.s.	0.507	0.168	0.311	0.928	0.020
ACTG2	49	16	120-162	0.469	0.918	n.s.	0.902	0.689	0.816	0.491	0.067
SW444	49	7	103-115	0.388	0.611	n.s.	0.572	0.214	0.392	0.368	0.103
Total								0.965*	0.995*	0.543	0.042

(b) *Ta*(b) *Tayassu pecari*											
SW1408	25	4	130-138	0.320	0.604	n.s.	0.530	0.184	0.333	0.475	0.084
SW857	25	5	130-140	0.480	0.747	n.s.	0.686	0.318	0.493	0.362	0.195
SW2411	25	10	186-228	0.520	0.873	n.s.	0.839	0.548	0.711	0.409	0.151
ACTG2	25	7	118-134	0.560	0.545	0.065	0.508	0.164	0.337	-0.028	0.097
SW444	25	3	98-106	0.320	0.594	n.s.	0.513	0.170	0.310	0.467	0.139
Total								0.826*	0.955*	0.351	0.139

Note: Number of analyzed individuals (N), number of alleles (NA), size of fragments (SIZE in bp), observed heterozygosity (H_o_), expected heterozygosity (H_e_), p (p-value), Polymorphic Information Content (PIC), probability of paternity exclusion (PE1 and PE2), inbreeding coefficient (Fis), and population structuring (Fst). n.s. (not significant at 5%). * (values of probability of exclusion combined).

## References

[Adegaetal2006] Adega F., Chaves R., Kofler A., Krausman P.R., Masabanda J., Wienberg J., Guedes-Pinto H. (2006). High-resolution comparative chromosome painting in the Arizona collared peccary (*Pecari tajacu*, Tayassuidae): A comparison with the karyotype of pig and sheep. Chromosome Res.

[Beck2006] Beck H. (2006). A review of peccary-palm interactions and their ecological ramifications across the Neotropics. J Mammal.

[Beiguelman1994] Beiguelman B. (1994). Dinâmica dos Genes nas Famílias e nas Populações.

[Botsteinetal1980] Botstein D., White R., Skolnick M., Davis R. (1980). Construction of a genetic linkage map in man using restriction fragment length polymorphisms. Am J Hum Genet.

[Bosmaetal2004] Bosma A.A., de Haan N.A., Arkesteijn G.J., Yang F., Yerle M., Zijlstra C. (2004). Comparative chromosome painting between the domestic pig (*Sus scrofa*) and two species of peccary, the collared peccary (*Tayassu tajacu*) and the whitelipped peccary (*T. pecari*): A phylogenetic perspective. Cytogenet Genome Res.

[DeutschandPuglia1990] Deutsch L.A., Puglia L.R. (1990). Os Animais Silvestres - Proteção, Doenças e Manejo.

[Excoffieretal1992] Excoffier L., Smouse P., Quattro J. (1992). Analysis of molecular variance inferred from metric distances among DNA haplotypes: Application to human mitochondrial DNA restriction data. Genetics.

[Excoffieretal2005] Excoffier L., Laval G., Schneider S. (2005). Arlequin ver 3.0: An integrated software package for population genetics data analyses. Evol Bioinform Online.

[Gemmelletal1997] Gemmell N.J., Allen P.J., Goodman S.J., Reed J.Z. (1997). Interspecific microsatellite markers for the study of pinniped populations. Mol Ecol.

[Gonela2003] Gonela A. (2003). Aplicação de marcadores microssatélites de *Sus scrofa domestica* na caracterização genética de populações de *Sus scrofa* sp (porco-Monteiro) e *Tayassu**pecari* (queixada) [PhD Thesis].

[Gongoraetal2002] Gongora J., Chen Y., Bernal J.E., Nicholas F.W., Moran C. (2002). Interspecific amplification of peccary microsatellite markers using porcine primers. Anim Genet.

[Lauetal2004] Lau J., Fernandez-Duque E., Evans S., Dixson A., Ryder O.A. (2004). Heterologous amplification and diversity of microsatellite loci in three owl monkey species (*Aotus azarai*, *A. lemurinus*, *A. nancymaae*). Conserv Genet.

[Limaetal2004] Lima J.F.S., Guedes F.B., Silva R.W., Hass I., Cavalli I.J., Silva J., Freitas T.R., Sbalqueiro I.J. (2004). Unexpected chromosomal alterations in *Tayassu tajacu* (Artiodactyla, Tayassuidae) in captivity. Braz J Vet Res Anim Sci.

[Lowdenetal2002] Lowden S., Finlayson H.A., Macdonald A.A., Downing A.C., Goodman S.J., Leus K., Kaspe I., Wahyuni E., Archibald A.I. (2002). The application of *Sus scrofa* microsatellite markers to wild suiformes. Conserv Genet.

[Marshalletal1998] Marshall T.C., Slate J., Kruuk L., Pemberton J.M. (1998). Statistical confidence for likelihood - Based paternity inference in natural populations. Mol Ecol.

[Medranoetal1990] Medrano J.F., Aesen E., Sharrow L. (1990). DNA extraction from nucleated red blood cells. Biotechniques.

[Nei1972] Nei M. (1972). Genetic distance between populations. Am Nat.

[Rohlf2000] Rohlf F.J. (2000). NTSYS-pc: Numerical Taxonomy and Multivariate Analysis System, v. 2.1.

[Slateetal1998] Slate J., Coltman D.W., Goodman S.J., Maclean I., Pemberton J.M., Williams J.L. (1998). Bovine microsatellite locos are highly conserved in red deer (*Cervus elaphus*), sika deer (*Cervus nippon*) and Soay sheep (*Ovis aries*). Anim Genet.

[SneathandSokal1973] Sneath P.H.A., Sokal R.R. (1973). Numerical Taxonomy.

[Tegelstrom1992] Tegelstrom H., Hoelzel A.R. (1992). Detection of mitochondrial DNA fragment. Molecular Genetic Analysis of Populations.

[WeirandCockerham1984] Weir B.S., Cockerham C.C. (1984). Estimating F-statistics for the analysis of population structure. Evolution.

[Wright1951] Wright S. (1951). The genetical structure of populations. Ann Eugenics.

