# Supplementary material for: Evaluation of genetic variability in the collared peccary Pecari tajacu and the white-lipped peccary Tayassu pecari by microsatellite markers
Source: Genet Mol Biol. 2010 Mar 1;33(1):62–7. doi: 10.1590/S1415-47572010005000002 (PMC3036067; doi:10.1590/S1415-47572010005000002)
Supplement: Table S1 — Localities and identification of voucher specimens. [file gmb-33-1-62-suppl1.pdf]

Table S1 - Localities and identification of voucher specimens

Pecari tajacu
(TIBAGI): Males: CAT6(32), CAT7(11), CAT9(33), CAT10(10), CAT16(60), CAT17(61), CAT18(62); Females - CAT2(02), CAT3(03), CAT4(05), CAT5(31), CAT8(34); (GUARAPUAVA): Male – CAT12(210), CAT13(211), CAT14(OPWN); Female - CAT11(WN), CAT15(WN); (FAZENDA RIO GRANDE): Male - CAT19(2088), CAT20(55373), CAT26(109311), CAT27(111239), CAT28(157372), CAT31(137632), CAT35(141624), CAT36(59445), CAT39(141606), CAT41(129004), CAT45(105609), CAT46(137186), CAT47(OPWN), CAT50(53977); Female - CAT21(56296), CAT22(52437), CAT23(118888), CAT24(144655), CAT25(159303), CAT29(127249), CAT30(124453), CAT32(158250), CAT33(130595), CAT34(144031), CAT37(53959), CAT38(127651), CAT40(165485), CAT42(1174), CAT43(61588), CAT44(131461), CAT48(61505), CAT49(62562). Cat = laboratory protocol; Numbers in parentheses = origin protocol; WN = without number.

Tayassu pecari
(GUARAPUAVA): Male - QUE2(WN), QUE3(251), QUE4(WN), QUE6(80), QUE7(546), QUE10(549), QUE12(551), QUE13(552), QUE15(263), QUE16(554), QUE17(55); Female - QUE5(099), QUE8(390), QUE14(553); (FAZENDA RIO GRANDE): Male - QUE19(413), QUE20(57341), QUE21(57549), QUE27(52705), QUE28(57093); Female - QUE22(56310), QUE23(56641), QUE24(53413), QUE25(53806), QUE26(53116), QUE29(62441). QUE = laboratory protocol; Numbers in parentheses = origin protocol; WN = without number.
